# Balancing Detection and Eradication for Control of Epidemics: Sudden Oak Death in Mixed-Species Stands

**DOI:** 10.1371/journal.pone.0012317

**Published:** 2010-09-14

**Authors:** Martial L. Ndeffo Mbah, Christopher A. Gilligan

**Affiliations:** Department of Plant Sciences, University of Cambridge, Cambridge, United Kingdom; University of California, United States of America

## Abstract

Culling of infected individuals is a widely used measure for the control of several plant and animal pathogens but culling first requires detection of often cryptically-infected hosts. In this paper, we address the problem of how to allocate resources between detection and culling when the budget for disease management is limited. The results are generic but we motivate the problem for the control of a botanical epidemic in a natural ecosystem: sudden oak death in mixed evergreen forests in coastal California, in which species composition is generally dominated by a spreader species (bay laurel) and a second host species (coast live oak) that is an epidemiological dead-end in that it does not transmit infection but which is frequently a target for preservation. Using a combination of an epidemiological model for two host species with a common pathogen together with optimal control theory we address the problem of how to balance the allocation of resources for detection and epidemic control in order to preserve both host species in the ecosystem. Contrary to simple expectations our results show that an intermediate level of detection is optimal. Low levels of detection, characteristic of low effort expended on searching and detection of diseased trees, and high detection levels, exemplified by the deployment of large amounts of resources to identify diseased trees, fail to bring the epidemic under control. Importantly, we show that a slight change in the balance between the resources allocated to detection and those allocated to control may lead to drastic inefficiencies in control strategies. The results hold when quarantine is introduced to reduce the ingress of infected material into the region of interest.

## Introduction

There is increasing interest in coupling epidemiological with economic models in order to identify optimal strategies for disease control [1]–[5]. Sethi [6] and others [7]–[9] first used optimal control theory to identify optimal strategies for disease control under a range of simplified epidemiological scenarios. More recent work has focused on introducing more realistic scenarios, for example when resources for control are limited [2], when disease occurs in heterogeneous landscapes [1], and when the time-scales for control occur within and across multiple seasons [3]. In this paper, we use these new approaches to address the problem of optimization of disease control in mixed species stands. We focus on a culling strategy, a widely used method for the control of plant and animal diseases in which infected hosts are removed to prevent further transmission of infection so that they are no longer capable of spreading infection [10]–[13]. Our principal objective is to identify optimal culling strategies for disease control and to investigate how limited resources should be balanced between disease detection and eradication in order to maximize the effectiveness of the control policy. Here, we define eradication in the sense frequently used in plant disease epidemiology as reducing the rate of production of inoculum during the course of the epidemic by destroying the sources of inoculum (culling) [14], [15].

We motivate our analyses for the control of a particular class of unidirectional epidemics in mixed two-species stands, in which both species are susceptible but one is a *spreader* and the other is an epidemiological *dead-end* to the pathogen cycle of infection. Such a scenario has been observed in the dynamics of diseases such as bubonic plague [16] in which rats are the *spreader* species, with humans being largely infected by the rat population [16]. Another example, which we study here, occurs in sudden oak death (SOD) in which the *spreader* may be an under-storey species, with the *dead-end* species frequently being a target for preservation [17], [18]. When the *dead-end* species is indeed targeted for preservation, a simple solution to the problem of disease control might be to eradicate the species driving the infection. Such a naïve solution is, however, far from optimal. Although it prevents further spread onto the target species, complete removal of the *spreader* species may have extremely negative impacts on the stability of the ecosystem. An optimal control strategy must seek to preserve both species. How this is done depends upon the growth and infection dynamics of the two host species, and importantly too on the ease with which infected *spreader* hosts are detected and removed.

Specifically, we consider the control of an epidemic of sudden oak death in Californian coastal forests, where the pathogen, an oomycete, (*Phytophthora ramorun*) mainly affects bay laurel (*Umbelluria californica*) - coast live oak (*Quercus agrifolia*) communities [10], [19]. The causal agent, *P. ramorun*, affects bay laurel that, in turn, acts as a source of inoculum for secondary infection. From infected bay laurel, the pathogen produces spores that spread aerially, by wind and rain splash dispersal mechanisms, to susceptible individuals (bay laurel and coast live oak)[10]. Bay laurel is an effective *spreader* species that seldom dies from infection. Coast live oak is only infected from bay laurel and dies from infection, accounting for millions of tree mortalities in California [10]. There is no transmission of infection from coast live oak but it is also primarily targeted for preservation. Several control methods have been tested to prevent and contain the spread of *P. ramorum* on bay laurel in Californian forests but culling of infected spreader trees and a quarantine policy to minimize introduction of inoculum are by far the most commonly used methods [18]. We consider a mixed species stand of bay laurel and coast live oak, in which the objective is to deploy a fixed amount of resource to preserve as many healthy trees of both species as possible, subject to placing a greater utility in preserving coast live oak than bay laurel. We show first that when there is a limit on expenditure, it is optimal to cull as many infected bay laurel trees as possible for SOD in two-species mixed evergreen communities. The result is unsurprising but our analyses yield considerably greater insight into the effectiveness of control strategies when allowance is made for incomplete knowledge of the infection status. The limited resource then needs to be partitioned into expenditure on detection as well as culling. In particular we investigate the trade-off in detection with eradication in achieving efficient disease control. Finally, we extend the results to consider how to optimize strategies that also include quarantine measures to reduce the ingress of infected material, for example by limiting access to forest.

### Model

A pair of systems of non-linear differential equations is used to describe the dynamics of an epidemic spreading on a community comprising two species, with unidirectional coupling and external infection. Control is applied to the system through culling of infected individuals and quarantine. These control measures respectively reduce the internal and external force of infection. By taking into account the economic costs attached to control strategies, we address the problem of disease control as a cost-effectiveness problem.

#### Epidemic Model

We consider a community in which a pathogen (*P. ramorum*) is able to infect two different host species. We assume that disease builds up on bay laurel, the *spreader* species, from which it spreads on to the coast live oak (*dead-end*), hereafter referred to as species 1 and 2 respectively. Each individual within the host community exists in one of the following states: susceptible (S) and infected (I). Since species 1 (*spreader*) is primarily targeted for control, its infected class can be further sub-divided into two sub-classes: infected and not yet detected (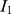) and infected and detected (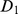). We also assume that the community is subject to an external source of infection due to free-living inoculum 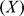 coming from outside the region of interest. The vital dynamics of each species are constrained by the carrying capacity of the environment, and the natural competition between species. Control is effected by culling involving constant removal of detected individuals from the species 1. The dynamics of the epidemic are given by the following set of differential equations:
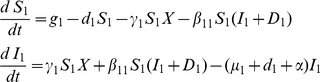
(1)

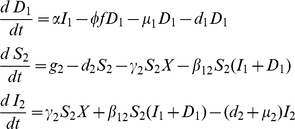
(2)where 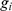 and 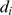 represent respectively the recruitment function and the rate of loss of each species, 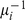 the infectious period, and 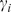 the rate of external infection with 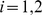. 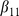 is the rate of infection within the first species, and 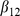 is the rate of infection from the first species to the second species; 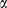 is the rate of detection of infected individual and 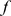 is the proportion of detected individuals that are culled. The parameter 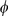 is the rate at which culled individuals are removed from the population. For the sake of simplicity, we assume that culled individuals are instantaneously removed from the population, giving 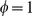. The model assumes homogeneous mixing (i.e., a spore originating from one individual is equally likely to land on and start infection on any other individual in the system). Given the scale of interest, namely a forest stand, this is a plausible assumption for *P. ramorum* which has the ability to spread readily by aerial dispersal of copiously produced spores over several kms.

For the sake of simplicity, we assume henceforth that the growth functions 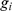 are given by the simple monomolecular function 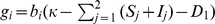 with 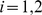; where 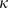 is the carrying capacity of the environment, and 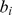 is the recruitment rate of each species. However, the results derived below hold for more complex functions, such as the commonly used logistic growth function. It is important to note that even though the disease dynamics on species 2 do not directly affect the behaviour of the epidemic on species 1, they do affect the vital dynamics of the second species which in return affect the influx of the first species.

#### Objective function

The criterion for optimization is to maximize the density of healthy individuals of both species, by controlling the culling rate subject to a budget constraint and differential utilities for species 2 over species 1. Hence we have to choose 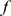 to maximize the integral
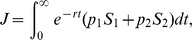
(3)under the propagation equations Eqs. 1–2 subject to the constraints of the epidemiological and economical system. Here, we denote the utility of species 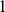 and 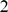, by 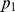 and 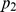, respectively: 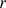 is a discount rate. The discount rate represents the rate the policy-maker is willing to pay to trade off the value of controlling today against the ensuing cost of increased infection in the future [20].

To solve the problem, we use an optimization approach based upon the Hamiltonian method [1], [2], which is a device for maximizing the objective function subject to economic constraints and the epidemiological dynamics of the model. Basically, we choose 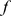 (the proportion of detected individuals culled per unit time) so as to maximize the current value of a mathematical expression known as the Hamiltonian, which takes into account the influence of the current infection and future evolution of disease as given by the state equations Eqs. 1–2 (see Methods). We assume that expenditure on detection and culling is constrained by a fixed budget (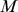) and is given by:
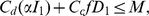
(4)where 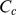 is the cost of culling per individual and 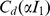 is the cost of detection per unit time. For a given detection strategy 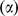, we assume that at a certain cost 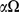 it would be possible to detect a very small number of infected individuals 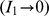. As the number of infected individuals (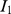) increases, the cost of detection decreases. This is justified by the fact that as the infected population becomes abundant, less marginal effort is required to detect additional infected individuals. Hence we define the cost of detection as 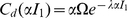, where 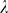 is the per capita rate at which the cost of detection 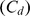 decreases. 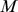 is the expenditure limit per unit time. The simple fixed budget constraint is used so as to encompass logistical limitations (e.g. finances and green waste disposal facilities) and for mobilisation and delivery of resources at the point of infection (e.g. trained personnel). In this paper we focus on the sensitivity of the outcome of the control strategy to the rate of detection, 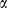. Given the way we define the cost of detection, it follows that changing the value of 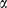 may be regarded as a surrogate for the effort expended on detection. For example, increased effort may involve visiting more sites and screening more trees within a site or increasing the amount of personnel-time deployed in detection.

#### Quarantine control

The effect of quarantine is implemented in combination with the culling strategy by reducing the rate of external infection (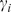 (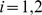)). In the case of sudden oak death, quarantine may be effected by reducing human-mediated dispersal by restricting access or by preventing import of potentially infected ornamental plants into designated regions at risk of disease. Control of this type is costly to implement and may also inflict indirect costs arising from restrictions on free circulation. Following [2], quarantine is introduced into the model by adjusting 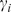, such that 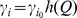 where 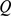 is the total amount of direct and indirect costs involved in the quarantine policy. We assume that 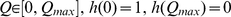. Thus, when there are no restrictions the rate of external infection 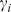 is equal to 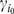, and when a total ban is imposed 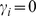 and the cost of restrictions is equal to 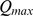. We assume that 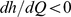 and 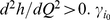 is then the value of 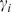 in the absence of quarantine. We choose 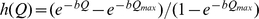 as, with 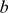 being a constant measuring the efficiency of the spending 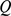. We also assume that the budget for quarantine is separated from the budget for detection and culling. The optimal strategy is now to choose 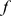 and 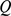 so as to maximize the integral
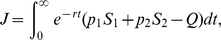
(5)subject to the same constraints as before plus an addition constraint 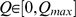 and 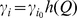. To solve the optimal control problem, we use the Pontryagin maximum principle [21] and follow the same procedure as with Eq. 3.

We first derive analytical solutions for the optimal strategies without quarantine by maximizing the objective function Eq. 3, subject to the epidemiological dynamics Eqs. 1–2 and the economic constraints (Eq. 4). Subsequently, we analyse the effects of changing the efficiency of detection on the effectiveness of control, with biologically plausible parameters for *P. ramorum* on bay laurel and coast live oak (Table 1). The scaled difference between the *area under the disease progress curve* (AUDPC) [22] for the epidemic with and without control is used as a measure to evaluate the efficiency of a given detection strategy on the effectiveness of control. The scaled difference between the AUDPCs is termed the *Difference in control*. We conclude our analysis by deriving an optimal solution when quarantine is used in combination with culling (Eq. 5).

**Table 1:** The values given here are used in numerical simulations unless stated otherwise.

Symbol	Description	Value
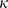	carrying capacity	3
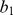	birth rate of bay laurel	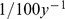
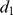	natural death rate of bay laurel	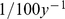
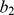	birth rate of coast live oak	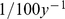
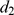	natural death rate of coast live oak	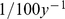
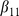	rate of infection from bay laurel to bay laurel	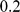
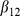	rate of infection from bay laurel to coast live oak	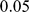
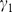	rate of primary infection on bay laurel	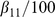
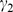	rate of primary infection on coast live oak	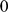
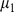	rate of death of bay laurel due to disease	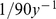
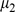	rate of death of coast live oak due to disease	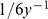
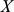	amount of external inoculum	0.01
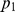	utility of bay laurel per individual per unit time	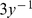
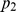	utility of coast live oak per individual per unit time	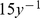
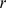	discount rate	0.05 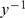
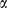	rate of detection of infected trees	varied
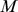	expenditure limit per unit time	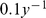
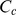	cost of culling per individual	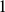

The epidemiological parameter values were derived from [28] and J.M.Davidson unpublished data. The relative magnitudes for the cost of culling and the utilities of bay laurel and coast live oak are expressed in arbitrary units.

## Results

The optimal strategy of control (see Methods) satisfies the following:
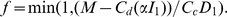
(6)We conclude that the optimal solution is to cull as many detected individuals as possible in species 1 (bay laurel). These results hold for all parameter values. We now analyse the quantitative effects of changing the detection rate 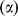 on the optimal solution.

**Figure 1:**
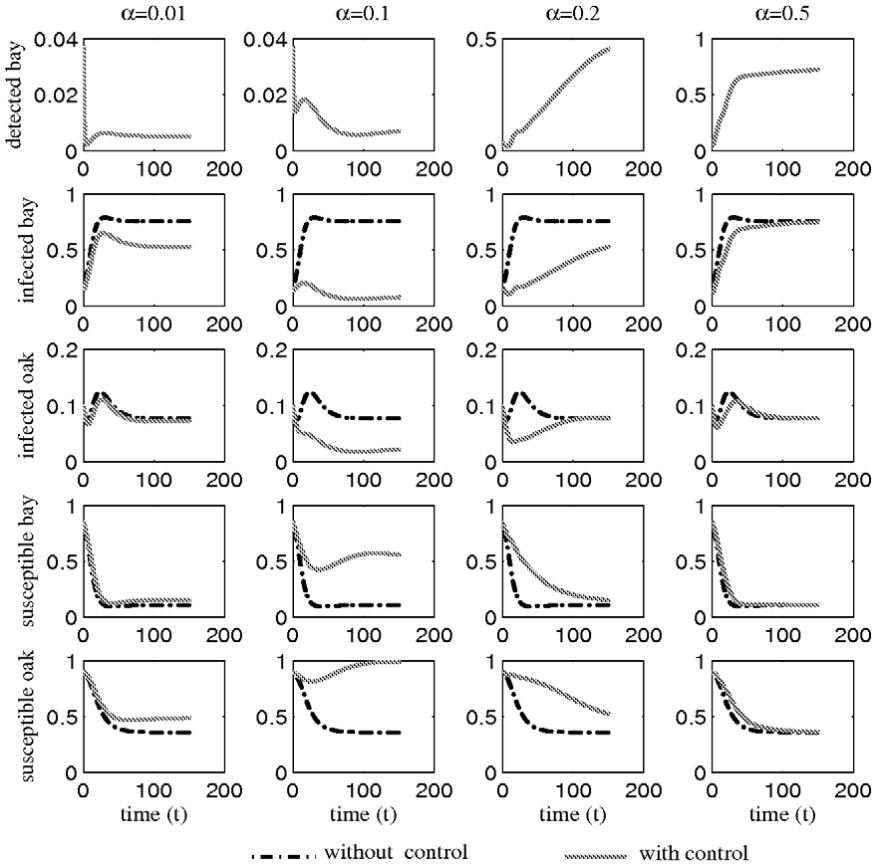
Dynamics of infection for different values of the detection rate (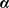). The dashed lines represent the dynamics without control, whereas the solid lines represent the dynamics under the optimal culling strategy. The figures are given respectively for 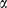 equal to 0.01, 0.1, 0.2 and 0.5 (from the left to the right). For 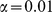 and 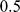, dynamics under control are almost identical to those without control.

Using the default parameter values given in Table 1, numerical simulations were carried out for different values of the initial density of infected trees and the rate of detection. At low detection rates, the disease dynamics under the optimal culling strategy are almost identical to those without control (see 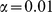 in Figs. 1). This is consistent with only a small proportion of infected individuals being detected when 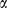 is low. It follows that a large proportion of infected individuals remain undetected throughout the epidemic. Only a small proportion of available resources are used for detection, leaving most of the available resources for culling. But because the majority of infected individuals remain undetected, culling has an insignificant effect on the dynamics of the epidemic even if 100% of detected individuals are culled at each unit of time.

For high detection rates, our simulations show that the culling strategy has little effect on the dynamics of infection. In fact, when an extensive detection strategy is used, for high values of 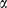, most of the infected individuals are detected over time, leaving only a small proportion of undetected sources of infection (see 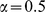 in Figs. 1). In this scenario, most of the available resources are used in detecting infected individuals and the remaining resources may just be enough to cull a small proportion of those individuals which have been detected (see 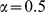 in Figs. 1). The remaining proportion of detected individuals that cannot be culled, due to the shortage of resources, continue to spread the infection. As with low detection rates, the dynamics of infection are scarcely affected by control.

The success of control strategies in preserving oaks (species 2) is remarkably sensitive to intermediate levels of detection efforts (c.f. 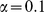 and 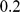 in Fig. 1). Above a certain value of 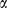, successful control is restricted to the early part of the epidemic but thereafter failing to bring the epidemic under control (c.f. infected oaks for 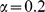 in Fig. 1). In this case, while there are sufficient resources to detect and remove a substantial proportion of detected bay laurel trees early on, the epidemic soon outstrips the resources available for control, negating the short-term advantage of a comparatively high expenditure on detection. We show, however, that when the detection level (and the associated cost) is reduced (c.f. 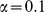), that the epidemic can be brought under control and a healthy population of oaks preserved (Fig. 1).

**Figure 2:**
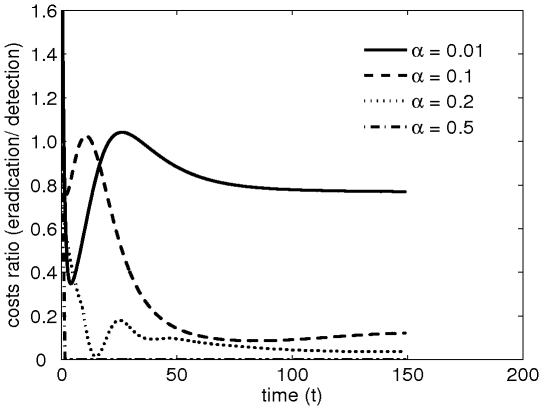
Ratio of costs of eradication to costs of detection for different values of the detection rate (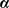). The ratios correspond respectively to the different scenarios presented in Fig. 1. For high levels of detection (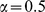), most resources are allocated to detection and almost none are left for eradication. For intermediate levels of detection (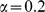 and 0.1), the short-term behaviour of the costs ratio is very sensitive to the value of the detection rate 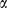.

It is intuitively appealing to enquire how the balance of costs for control and detection change during the course of an epidemic. We show this in Fig. 2 for different values of 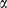. While long-term trends are apparent, simple interpretation of the early dynamics for cost (cf oscillations in Fig. 2) is not straightforward. The particular dynamics depend not only on the initial conditions but also on the interactions between the functional relationships of the costs (Eq. 4) and the underlying disease dynamics. Nevertheless, the principal result of our analyses (Fig. 1) shows clearly the importance of selecting intermediate levels of detection in efficient management of disease under fixed budgets.

**Figure 3:**
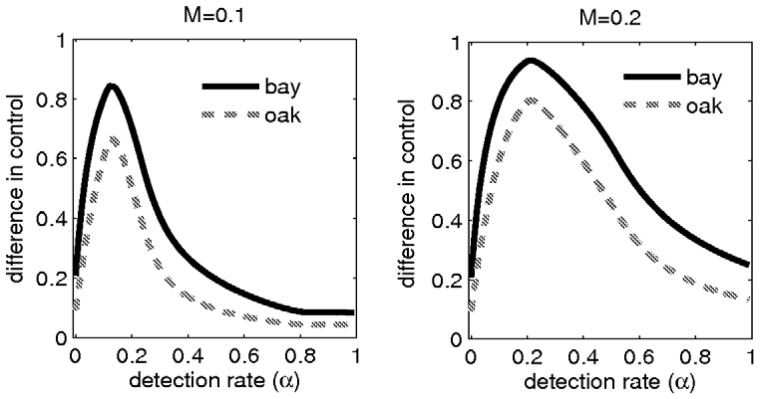
*Difference in control* on bay laurel and coast live oak for different disease monitoring strategies (detection rates). The difference in control is defined as to be the scaled value of the difference between the area under disease progress curves for the epidemics without and with control. The range of optimal disease monitoring strategies increase with the expenditure limit (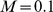 *vs* 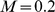).

The optimal choice of 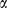 depends upon the value of the expenditure limit 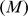. It cannot be derived analytically. Numerical simulations show that the range of values of 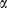 for which the control strategy has a large positive effect on the dynamics of infection (measured by the difference in control) increases with the budget. The optimal value for 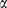 is deflected to the left, i.e. decreases as 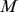 decreases (Fig. 3). Moreover, for a given value of the expenditure limit (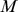), there exists a threshold value for the detection rate (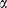) above which the control strategy fails to bring the epidemic under control.

Our results show that the trade-off between the cost (and efficiency) of detection and the cost of culling is an important factor that must be taken into account for optimal use of resources, when as is usually the case, there are budgetary constraints.

### Quarantine control

Now, we suppose that in addition to culling of infected bay laurel, the transmission rate, 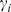 (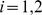) can be altered by imposing a quarantine control that restricts the rate of entry of external infection. The optimal solution is obtained by selecting 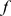 and 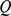 in the objective function Eq. 5. When 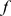 is given by Eq. 6, the optimal value 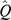 is given by
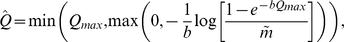
(7)where 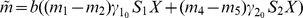, and 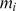 are co-state variables defined in the Methods.

**Figure 4:**
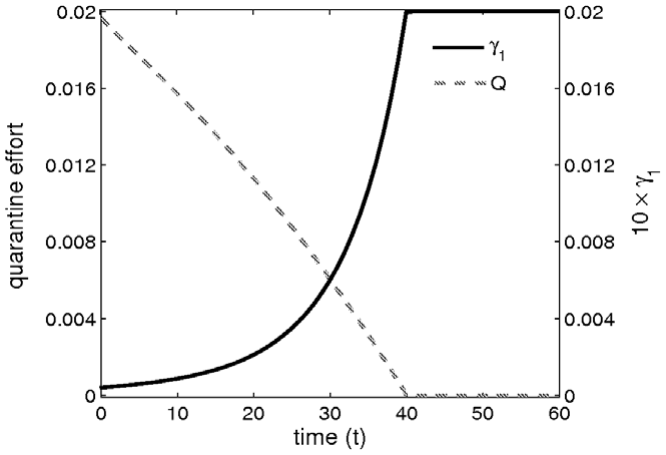
Optimal quarantine effort and corresponding value of 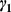. The value of 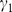 is multiplied by 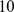, for convenience in matching the scale of 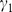 to that of 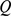. Default parameters values were used, except 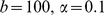 and 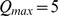.

Using the default parameter values given in Table 1, numerical simulation shows that it is not always optimal to apply quarantine. The decision to implement quarantine or not is a function of the efficiency of the quarantine measures, and of the level of external inoculum that enters the system (Fig. 4). The monotonic behaviour of the optimal quarantine strategy is a direct consequence of the assumption of a constant rate of entry of external inoculum Eqs. 1 and 2. The time at which it is no longer optimal to apply quarantine is delayed as the amount of external inoculum 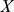 increases, and decreases with increasing 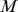 (results not shown).

Numerical simulation shows that, for small values of 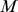 it is not possible to bring an epidemic under control, regardless of the efficiency of the quarantine measures. This is consistent with the assumption that disease increase is mainly driven by the internal forces of infection. We conclude that it is therefore not optimal to apply quarantine For small values of 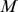.

## Discussion

We have used an SI-X metapopulation model to describe the dynamics of an epidemic spreading on a two-species host community in which there is a spreader host (bay laurel) and a target species that we wish to preserve (coast live oak). A combination of control theory with an epidemiological model, enabled us to identify optimal strategies for the detection and control of the pathogen (*P. ramorum*) in order to preserve the target species within the community. We first considered a simple culling strategy directed at the detection and removal of symptomatic plants from the spreader species.

In contrast with many previous analyses for optimal control of disease [3], [5], [8], we assume that resources for control are limited. We also assume that expenditure for disease detection (sampling) and control (culling) are drawn from the same funds. In considering culling in the absence of quarantine, we have proved, analytically, that the optimal culling strategy involves removal of as many detected individuals as possible, in the spreader (bay laurel) species driving the epidemic. The efficiency of the optimal culling strategy in bringing the epidemic under control depends upon a careful balancing of resources for disease detection and for culling (Figs. 1 and 3). We show, in particular, that both high and low detection rates fail to bring the epidemic under control. Successful control, in terms of maximizing the amounts of susceptible bay laurel and coast live oak, is more likely to be achieved at intermediate levels of detection (Fig. 1). The optimal level of detection depends upon the value of the expenditure limit (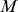) (*cf* Fig. 3). The addition of quarantine to the control strategy serves to reduce human-mediated dispersal of inoculum into the region of interest. Our results suggest, however, that priority should still be given to the culling strategy (detection and culling) rather than quarantine in the allocation of the budget for epidemics in which most spread is driven by secondary infection within the region of interest. The current analysis holds for a spatial structure for stand size of the order of several kms in which most infection occurs by secondary transmission within the stand. Analyses for larger scales, could naturally be addressed using a metapopulation framework [23] in which sub-populations represent stands with some transmission of infection occurring amongst stands. We anticipate that the role of quarantine would acquire greater importance in this situation.

Surprisingly little attention has previously been given to optimization of control strategies that take account of costs and benefits for detection and control of infected hosts. Previous work, has focused on the control of invading species, exemplified by gypsy moth *Lymantria dispar* [14]. Bogich et al. [14] demonstrated the importance of incorporating the trade-off between detection and eradication in models of invasive species control. But, the analyses were done without taking account of the temporal dynamics of colony distribution of the pest. Hence Bogich et al. [14], address the problem of resource allocation as a one time allocation which does not allow reallocation of resources in response to the temporal dynamics. The approach is analogous to the identification of treatment efforts that are designed to reduce the basic reproductive number (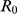) below one for a pest or pathogen. While such an approach may be effective in preventing an epidemic or pest outbreak, it is not necessarily economically optimal in terms of matching the treatment effort with changing infection pressure reflected in the transient dynamics of the pest or pathogen. Hence Zaric and Brandeau [24] show that allowing for reallocation of funds may generate more health-benefit than strategies based upon a fixed (one-time) allocation of resources.

In applying our model to the spread of sudden oak death in a plant community typical of mixed evergreen forests in coastal California, our analyses were designed to identify optimal culling and quarantine strategies to preserve as much as possible of the spreader species (bay laurel) and especially the target (coast live oak) species within the community. The objective is based upon three major concerns. Firstly, the threat of *P. ramorum* is more pronounced on oak trees than other species [10], [25]. Secondly, oak trees promote greater biodiversity within forest communities than bay laurel [10]. Lastly, in many areas, especially close to conurbations such as San Francisco, coast live oak is considered to have greater aesthetic and conservation value than bay laurel. The balance of expenditure on detection and treatment for disease management, however, applies to a very wide range of practical disease control problems. Although our analyses have been motivated for a specific host-pathogen system, the methodology is generic and can be applied to a wide range of host pathogen systems in which budgets have to be allocated to detection and control. We postulate that intermediate levels of detection are likely to prove optimal for many of these.

Several assumptions were used in the derivation of the model and execution of the analyses. Foremost amongst these are the epidemiological assumptions that the rates of infection are constant over time and that culling occurs without delay after detection of symptomatic hosts. The methods can easily be adapted to allow, for example, for temporal forcing due to seasonal variations typified by the spread of *P. ramorum*, which is mainly driven by seasonal factors such as rainy seasons and EL Niño [17], [18]. Preliminary analysis suggests that accounting for temporal forcing does not change the qualitative nature of the results. Future work will consider first, the effect of logistical delays between detection and culling, as well as adjustments in the detection rate as a response to disease progression and control.

## Methods

To maximize the objective function
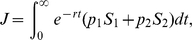
(8)subject to the disease dynamics equations, we use the Pontryagin maximum principle [21] which is commonly used to address problems of optimal control for continuous state system [3], [5], [26]. This is done by optimizing the current value of the Hamiltonian as given by
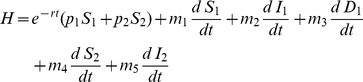
(9)where 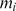 (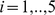), the costate variables, satisfy the following system of differential equations
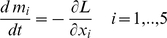
(10)where 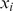 is the state variable corresponding to 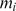. Because of the presence of the constraint
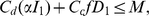
(11)the standard procedure requires the introduction of a Lagrangian defined as
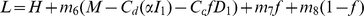
(12)where 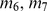 and 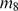 are Lagrangian multipliers which satisfy the complementarity slack conditions [21]

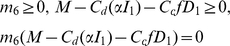
(13)

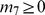
 and 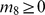. The first order conditions for an optimum require that
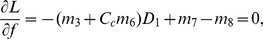
(14)along the trajectory of an optimal solution, and 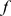 being chosen such as to maximize the Hamiltonian. From the maximum condition on the Hamiltonian, it follows that
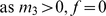


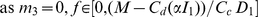


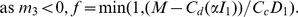



As the rate of detection is taken to be constant over time, there is a positive correlation between the inflow of infected individuals (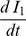) and that of detected individuals (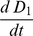). Given that culling is restricted to detected individuals 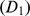, it is therefore natural that the optimal condition on 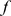 (culling strategy) depends on 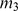 (the marginal benefit of increasing the stock of detected individuals (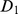)).

### Interior solution

Consider a path which satisfies the first order conditions above, and suppose that
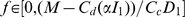
(15)over an open segment of this path. Within this segment it must be the case that 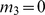. By differentiating 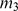 over that open segment, it follows that 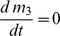. Hence from Eq. 10, we have
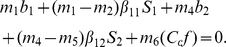
(16)From an economical view point, the costate variables can be interpreted as shadow prices. Where variables 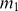 and 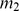 indicate respectively the marginal benefit to society of increasing the stock of susceptible individuals (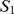) and infected individuals (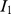) of the first species by one unit [5], [27]; and 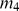 and 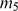 are marginal benefits from the second species. Because infection is harmful, and increasing the stock of infected individuals will result in decreasing the stock of susceptible individuals, the shadow prices 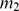 and 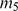 are negative. It then follows that we have 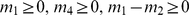 and 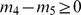. From the complementary slack conditions, 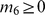. It follows that the left hand side of Eq. 16 is positive. Therefore Eq. 16 is satisfied if and only if 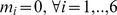 on the open interval. But since 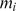 is a solution of Eq. 10, and 
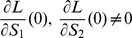
, it follows that one cannot have 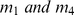 equal to 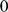 on an open interval. Hence, we conclude that there is no path satisfying the first order conditions for an optimum, for which 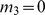 on an open interval.

### Optimal solution

From the above results, it follows that an optimal trajectory for the control variable 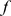 is either given by 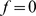,
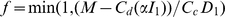
(17)or a switch strategy between 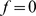 and 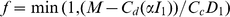. Given that 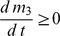, an optimal switching strategy can only be a single switch from
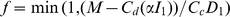
(18)to 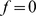.

Extensive numerical simulation shows that the optimal trajectory of the control variable is given by
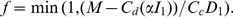
(19)This can be justified by the fact that there is a constant inflow of pathogen from sources external to the community. Therefore, having 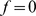 would give free course for disease to build up within the community, and subsequently generating a new outbreak.

### Quarantine

The Hamiltonian for the case with quarantine is the same as Eq. 9 except for 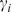 which are replaced by 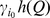 and the objective function which is given by Eq. 5. Analysis shows that the optimal culling strategy is still given by
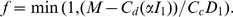
(20)With the additional constraint that the quarantine variable 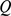 is selected from the set 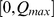 so as to maximize the Hamiltonian, taking all other variables as given. The optimal value of 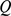 is thus equal to
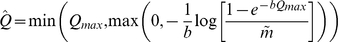
(21)where 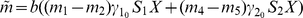.
